# Characterization of Gramicidin A in Triblock and Diblock Polymersomes and Hybrid Vesicles via Continuous Wave Electron Paramagnetic Resonance Spectroscopy

**DOI:** 10.3390/biomimetics10030154

**Published:** 2025-03-02

**Authors:** Emma A. Gordon, Indra D. Sahu, Joel R. Fried, Gary A. Lorigan

**Affiliations:** 1 Materials Science and Technology Division, Los Alamos National Lab, Los Alamos, NM 87545, USA; egordon@lanl.gov; 2 Natural Science Division, Campbellsville University, Campbellsville, KY 42718, USA; idsahu@campbellsville.edu; 3 Department of Chemistry and Biochemistry, Miami University, Oxford, OH 45056, USA; gary.lorigan@miamioh.edu; 4 Department of Chemical Engineering, University of Louisville, Louisville, KY 40292, USA

**Keywords:** membrane mimetics, triblock and diblock polymersomes, hybrid vesicles, gramicidin A, electron paramagnetic resonance spectroscopy

## Abstract

Studying membrane proteins in a native environment is crucial to understanding their structural and/or functional studies. Often, widely accepted mimetic systems have limitations that prevent the study of some membrane proteins. Micelles, bicelles, and liposomes are common biomimetic systems but have problems with membrane compatibility, limited lipid composition, and heterogeneity. To overcome these limitations, polymersomes and hybrid vesicles have become popular alternatives. Polymersomes form from amphiphilic triblock or diblock copolymers and are considered more robust than liposomes. Hybrid vesicles are a combination of lipids and block copolymers that form vesicles composed of a mixture of the two. These hybrid vesicles are appealing because they have the native lipid environment of bilayers but also the stability and customizability of polymersomes. Gramicidin A was incorporated into these polymersomes and characterized using continuous wave electron paramagnetic resonance (CW-EPR) and transmission electron microscopy (TEM). EPR spectroscopy is a powerful biophysical technique used to study the structure and dynamic properties of membrane proteins in their native environment. Spectroscopic studies of gramicidin A have been limited to liposomes; in this study, the membrane peptide is studied in both polymersomes and hybrid vesicles using CW-EPR spectroscopy. Lineshape analysis of spin-labeled gramicidin A revealed linewidth broadening, suggesting that the thicker polymersome membranes restrict the motion of the spin label more when compared to liposome membranes. Statement of Significance: Understanding membrane proteins’ structures and functions is critical in the study of many diseases. In order to study them in a native environment, membrane mimetics must be developed that can be suitable for obtaining superior biophysical data quality to characterize structural dynamics while maintaining their native functions and structures. Many currently widely accepted methods have limitations, such as a loss of native structure and function, heterogeneous vesicle formation, restricted lipid types for the vesicle formation for many proteins, and experimental artifacts, which leaves rooms for the development of new biomembrane mimetics. The triblock and diblock polymersomes and hybrid versicles utilized in this study may overcome these limitations and provide the stability and customizability of polymersomes, keeping the biocompatibility and functionality of liposomes for EPR studies of membrane proteins.

## 1. Introduction

Most commonly used membrane mimetic systems have shortcomings that limit their use in membrane protein studies. Membrane proteins play an important role in many biological systems and functions [1]. Given that membrane proteins are critical to biological systems, it is important to study their structure and function in a native state. Detergent micelles, bicelles, and liposomes are frequently used membrane mimetics [2]. Detergent micelles, formed with short-chain detergents, are used to solubilize membrane proteins but are an inaccurate representation of a lipid bilayer [3]. Bicelles consist of a mixture of both long-chain lipids and short-chain detergents to form a disc-like bilayer. Bicelles are limited by the composition of lipids and detergents and therefore are often incompatible with the membrane protein of interest [4,5,6]. Liposomes, although they accurately mimic the bilayer, can be heterogenous in size and shape, which may cause protein misfolding [7]. Recently, triblock and diblock polymersomes and hybrid vesicles have been used as biomimetics to overcome these limitations [8].

Polymersomes, or polymer vesicles, are composed of amphiphilic block copolymers and form a bilayer with a hydrophobic core [9]. They are well suited for biochemical and biophysical applications due to their potential in drug delivery, artificial organelles, and gene therapy [10,11]. Polymersomes are more stable than liposomes and can be customized both chemically and physically [12]. They closely mimic native membrane conditions and support the incorporation of large and complex membrane proteins, including multi-subunit assemblies, without compromising their stability or activity, facilitating high-throughput approaches for membrane protein characterization [13]. These properties make them attractive membrane mimetics for proteins that are otherwise incompatible with other commonly used membrane mimetics. This work used diblock, poly(2-methyl-2-oxazoline)_6_-poly(dimethylsiloxane)_17_ (PMOXA_6_-PDMS_17_), and triblock, poly(2-methyl-2-oxazoline)_6_-poly(dimethylsiloxane)_35_-poly(2-methyl-2-oxazoline)_6_-(PMOXA_6_-PDMS_35_-PMOXA_6_), copolymers to form polymersomes. PMOXA is a chemically stable amorphous polymer that is soluble in aqueous solutions and makes up the hydrophilic unit of the system [14]. PDMS is a flexible and inert polymer that makes up the hydrophobic unit of the system.

Hybrid vesicles are a blend of phospholipids and block copolymers with a membrane composition containing both components [15]. They have both the stability and the customizability of the polymersomes but keep the biocompatibility and functionality of liposomes [16]. Although in its infancy, the interest in hybrid vesicles lies in drug delivery and tuning the membrane for specific targets [17]. Functionalization of membrane surface, fluidity, and stimuli responsiveness are all properties of hybrid vesicles that can be adjusted based on the need of the protein of interest, making hybrid vesicles useful membrane mimetics [18,19]. In this work 1,2-dimyristoyl-sn-glycero-3-phosphocholine (DMPC), dipalmitoylphosphatidylcholine (DPPC), 1-palmitoyl-2-oleoyl-sn-glycero-3-phosphocholine (POPC), and dipalmitoylphosphatidylcholine DOPC lipids were used along with diblock and triblock copolymers to form hybrid vesicles.

Gramicidin A is a very-well-characterized peptide [20]. It is part of a family of antibiotics produced by the bacterium *Bacillus brevis* [21]. Gramicidin A is a short peptide composed of 15 amino acids that form α-helices. The hydrophobic peptide sits inside the lipid bilayer and can incorporate as a monomer or as a head-to-head dimer [20]. The interest in gramicidin comes from its ability to form ion channels, specifically, its ability to form monovalent cation conducting channels [22]. This work incorporates gramicidin A in both polymersome and hybrid vesicle biomimetic membranes.

EPR spectroscopy is a powerful technique in biophysical membrane studies. It can be used to study membrane protein’s structure and dynamics through the use of a free radical spin label [23,24,25]. EPR spectroscopy has been used to characterize gramicidin A previously [26,27]. Both spin-labeled lipids and spin-labeled peptides have been used alongside EPR to study the behavior and conformation of gramicidin A in a lipid membrane [26,27,28]. Studies of peptide aggregation [29,30,31], dimer formation [26], mono- and double-spin-labeled behavior [28], and structural changes [32] have been investigated using EPR spectroscopy and molecular dynamic simulations [33]. However, these studies have been limited to gramicidin A in a lipid membrane environment.

Studies of gramicidin A in a polymersome or hybrid vesicle environment have been limited to ion conduction experiments [14,21,22,29,34]. Patch clamps, fluorescent probes, micro-fabricated arrays, and capacitance experiments were conducted. These studies focused on the function and capacitance of gramicidin A in thicker polymer membranes, but lacked structural characterization of the peptide inside the polymersomes. Understanding how the dynamic motion of gramicidin A behaves in these mimetic systems can provide ideas on the designing of better platforms for studying more complex membrane proteins. This study will provide a foundational understanding of how triblock and diblock polymersomes, as well as hybrid vesicles, impact protein–mimetic interactions [13]. Additionally, this study will highlight the potential of these systems to support proper functional peptide incorporation for biophysical studies [35].

In this study, gramicidin A was incorporated into both polymersomes and hybrid vesicles and characterized by CW-EPR spectroscopy and transmission electron microscopy (TEM). Triblock (ABA) and diblock (AB) polymersomes and hybrid vesicles were used as biomimetic membranes. CW-EPR lineshape analysis was carried out, revealing line broadening in the polymersome and hybrid vesicle samples when compared to the liposome samples. This is consistent with the structural changes to thicker membranes with an incorporated membrane protein, as proposed by previous studies [36].

## 2. Materials and Methods

### 2.1. Polymersome Preparation

The diblock and triblock polymers were purchased from Polymer Source, Inc. The diblock had block lengths of 6 PMOXA segments and 17 PDMS segments (PMOXA_6_-PDMS_17_), an *M*_W_ of 1300-*b*-500, and a polydispersity of 1.2 using a benzyl linker, part number P10649-DMSMEOXZ. The triblock composition was PMOXA_6_-PDMS_35_-PMOXA_6_, *M*_W_ of 500-*b*-2600-*b*-550 with a propyl linker, and a polydispersity of 1.3 (part number P14521D-MOXZDMSMOXZ).

Polymersomes were prepared from either triblock PMOXA_6_-PDMS_35_-PMOXA_6_ or diblock PMOXA_6_-PDMS_17_ copolymers via a thin film method [34]. To make the film, 5 mg of polymer was dissolved in 1 mL of ethanol and evaporated under nitrogen to form a thin film around a pear-shaped flask. The sample was dried overnight in a desiccator. The film was rehydrated using a buffer containing 100 mM NaCl and 20 mM HEPES at a pH of 7.0 to a final concentration of 100 mM. Polymersomes then underwent several freeze/sonication cycles.

### 2.2. Hybrid Vesicle Preparation

Block copolymer solutions were prepared from either triblock PMOXA_6_-PDMS_35_-PMOXA_6_ or diblock PMOXA_6_-PDMS_17_ copolymers. Lipid solutions were made with either DOPC, POPC, DPPC, or DMPC powdered lipids. The hybrid vesicles were formed via a thin film method [18]. To make the film, 5 mg of polymer was dissolved in 1 mL of ethanol, combined with a 20 mg/mL chloroform solution of lipids, and evaporated under nitrogen to form a thin film around a pear-shaped flask. The sample was dried overnight in a desiccator. The film was rehydrated in a buffer containing 100 mM NaCl and 20 mM HEPES at a pH of 7.0 to a final concentration of 100 mM. Polymersomes then underwent several freeze/sonication cycles.

### 2.3. Vesicle Preparation

Vesicle samples were composed of either DOPC, POPC, DPPC, or DMPC. Powdered lipids were dissolved in chloroform and evaporated to form a thin film on the side of a heart-shaped flask [37]. The sample was dried overnight in a vacuum desiccator. The lipid film was suspended in a buffer containing 100 mM NaCl and 20 mM HEPES at a pH of 7.0 to a final concentration of 100 mM. The solution was vortexed vigorously to mix completely and vesicles were spontaneously formed, resulting in a homogeneous milky solution after 3 freeze/sonication cycles. Dynamic light scattering experiments were used to confirm the size of the vesicles.

### 2.4. Spin-Labeled Lipids

The 16:0 5-doxyl PC, 16:0 12-doxyl PC, and 16:0 16-doxyl PC lipids were purchased from Avanti Polar Lipids. Vesicles and polymersomes were prepared as described above, but 1 mol% of lipid spin label was added to the mixture before drying into a thin film under nitrogen gas.

### 2.5. Transmission Electron Microscopy

Transmission electron microscopy (TEM) is a powerful imaging technique that uses a beam of electrons to visualize and determine the size and shape of biomolecules at the atomic or molecular level. All grid samples were examined in a JEOL JEM-1200EX II TEM instrument. Sample preparation, TEM imaging, and image analysis were completed at the Center for Advanced Microscopy and Imaging at Miami University.

### 2.6. Gramicidin A Preparation

The peptide gramicidin A was synthesized via optimized Fmoc solid-phase peptide synthesis (SPSS) [38]. Peptide synthesis was conducted on a CEM Liberty Blue synthesizer equipped with a Discovery Blue microwave system. The mutants A3C and W13C were synthesized in order to attach the spin label MTSL (S-(1-oxyl-2,2,5,5-tetramethyl-2,5-dihydro-1H-pyrrol-3-yl)methyl methanesulfonthioate) to the cysteine. Spin labeling was followed by HPLC purification and incorporation into the polymersomes via a thin film method. The peptide was dissolved in DMSO:EtOH 1:1 at 0.2 mg/mL and added to predissolved polymersomes or vesicles in a pear shaped flask. The solvent was evaporated by N_2_ gas, making a uniform thin film inside the flask. It was left to dry in a desiccator overnight to remove any remaining solvent. The film was rehydrated with a 10 mM HEPES buffer at 7.4 pH. Following rehydration, the solution went through 5 freeze–thaw cycles with intermittent vortexing to help to maintain a homogenous mixture. The protein-to-lipid ratio was set to 1:400.

### 2.7. CW-EPR Measurements

EPR experiments were conducted at the Ohio Advanced EPR Laboratory. CW-EPR spectra were collected at the X-band on a Bruker EMX CW-EPR spectrometer using an ER041xG microwave bridge and ER4119-HS cavity coupled with a BVT 3000 nitrogen gas temperature controller. Each spin-labeled CW-EPR spectrum was acquired with a central field of 3433 G and sweep width of 150 G, modulation frequency of 100 kHz, modulation amplitude of 1 G, and microwave power of 10 mW at room temperature.

### 2.8. EPR Spectral Simulations

EPR spectral simulations were performed using the non-linear least squares (NLSL) program with the macroscopic order microscopic disorder (MOMD) model developed by the Freed group [39,40] following similar simulation conditions reported in the literature [41]. The principle components of the hyperfine interaction, tensor A = [6.7 ± 0.5, 5.7 ± 0.5, 33.4 ± 0.8] G and g-tensors g = [2.0080 ± 0.0002, 2.0062 ± 0.0004, 2.0026 ± 0.0001] for site 1 and A = [5.3 ± 0.5, 6.7 ± 0.5, 34.8 ± 0.8] G and g-tensors g = [2.0081 ± 0.0002, 2.0063 ± 0.0004, 2.0023 ± 0.0001] for site 2, were obtained from the literature and tightly refined to obtain the best fit from the EPR spectra. The rotational diffusion tensors were varied while simulating the corresponding EPR spectra. Rigid/slower and higher/faster motional components of the EPR spectrum were accounted for in a two-site component fit procedure. The best fit rotational correlation times and the relative population of both components were determined using the Brownian diffusion model.

## 3. Results and Discussion

**Scheme 1:**
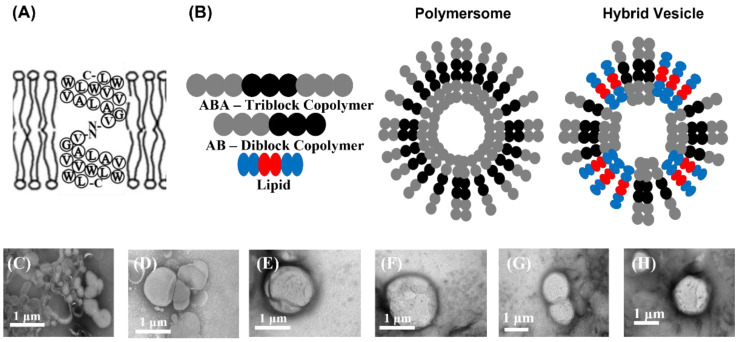
(**A**) Schematic of gramicidin A in a lipid bilayer in a head-to-head dimer formation. (**B**) Schematic of polymersomes and hybrid vesicles. TEM micrographs of (**C**) liposomes composed of DMPC lipids, (**D**) liposomes composed of DPPC lipids, (**E**) polymersomes composed of triblock copolymers, (**F**) polymersomes composed of diblock copolymers, (**G**) hybrid vesicles composed of POPC and triblock copolymers, and (**H**) hybrid vesicles composed of POPC and diblock copolymers.

Scheme 1A shows the structure of gramicidin A incorporated into a lipid bilayer in a head-to-head dimer formation. In this study, two mutants were used, A3C and W13C, both located inside the membrane. Scheme 1B depicts both polymersomes and hybrid vesicles composed of triblock copolymers, diblock copolymers, and lipids. In this study, the triblock PMOXA_6_-PDMS_35_-PMOXA_6_, diblock PMOXA_6_-PDMS_17_, and lipids DOPC, POPC, DPPC, and DMPC were used to form the polymersomes and hybrid vesicles. The thickness of the block copolymer membrane ranges from about 9 to 12 nm while a traditional liposome membrane thickness is about 5 nm [33]. The size and shape characterization of the vesicles and polymersomes can be seen in Scheme 1C–H. TEM micrographs of liposomes composed of DMPC and DPPC are shown in Scheme 1C,D, respectively. Polymersomes composed of the triblock and diblock copolymers are shown in Scheme 1E,F, respectively. Lastly, TEM micrographs of hybrid vesicles composed of both POPC and either triblock or diblock copolymers are shown in Scheme 1G,H, respectively.

**Figure 1:**
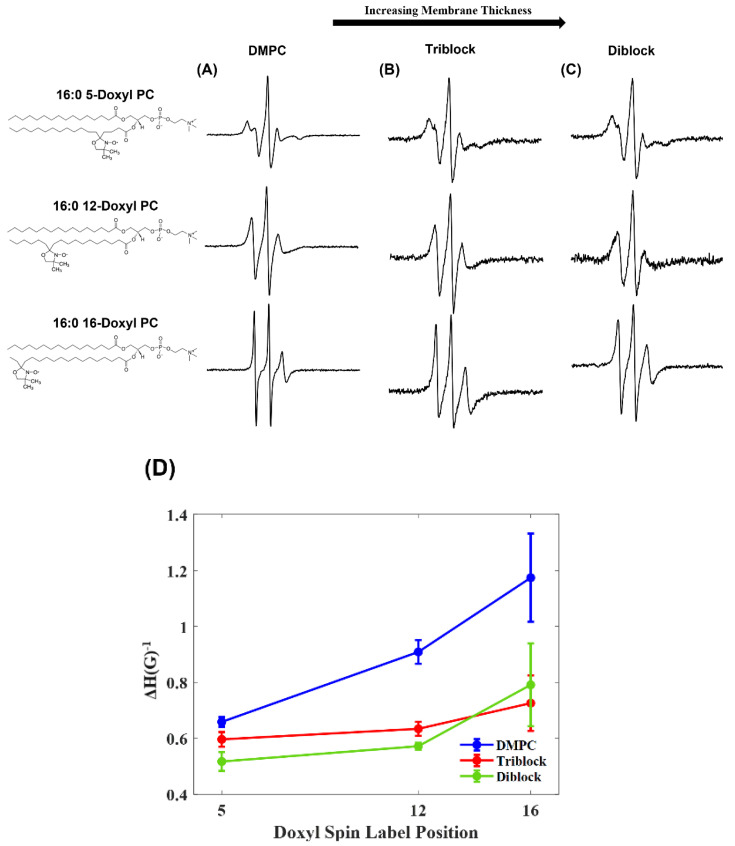
CW-EPR spectra of spin-labeled lipids incorporated into liposomes and polymersomes collected at 298 K. (**A**) Spectra for liposomes composed of DMPC with incorporated 5-, 12-, 16-doxyl PC spin-labeled lipids. (**B**) Spectra for polymersomes composed of triblock copolymers with incorporated 5-, 12-, 16-doxyl PC spin-labeled lipids. (**C**) Spectra for polymersomes composed of diblock copolymers with incorporated 5-, 12-, 16-doxyl PC spin-labeled lipids (**D**) Inverse central linewidth (ΔH^−1^) of EPR spectra as a function of the spin label lipid incorporated in the vesicles.

Previous studies using fluorescence spectroscopy have shown that polymersomes are more rigid, less fluid, and have higher lateral pressure within the bilayer than traditional liposomes [12]. This has been attributed to both the size and thickness of the membranes as well as the polymer structure. CW-EPR spectroscopy was used to investigate polymersomes using spin-labeled lipids 16:0 5-doxyl PC, 16:0 12-doxyl PC, and 16:0 16-doxyl PC. Figure 1 shows the CW-EPR spectra for liposomes composed of DMPC as well as polymersomes composed of the triblock copolymer and the diblock copolymer with the incorporated spin-labeled lipids. Samples containing 16-doxyl PC showed the sharpest peaks. The 16-doxyl PC is situated deep inside the membrane; the further into the hydrophobic core the spin label is located, the sharper the peaks would be as the spin label has more space to move near the bottom of the acyl chain. In contrast, samples containing 5-doxyl PC showed the broadest peaks when compared to 12- and 16-doxyl PC. The 5-doxyl PC is located closer to the polar headgroups of the membranes and has more restricted motion.

A trend was also seen between the liposomes and the polymersomes. Differences in lineshape and linewidth were observed between DMPC liposomes, triblock polymersomes, and diblock polymersomes. As the membrane thickness increased, so did the linewidth of the CW-EPR spectra. This increase in membrane thickness also increases the lateral pressure in the bilayer, which would cause the spin label to have more rigid motion, resulting in broader peaks in polymersome samples. In order to quantify the broadening in the spectra, the inverse central linewidth was calculated from the CW-EPR spectra shown in Figure 1A–C. The inverse central linewidth gives a quantitative measure of the side-chain mobility of the spin label [42]. Figure 1D shows an increase in the inverse central linewidth value the further the spin label is positioned into the membrane, indicating a sharper peak in the spectra.

**Figure 2:**
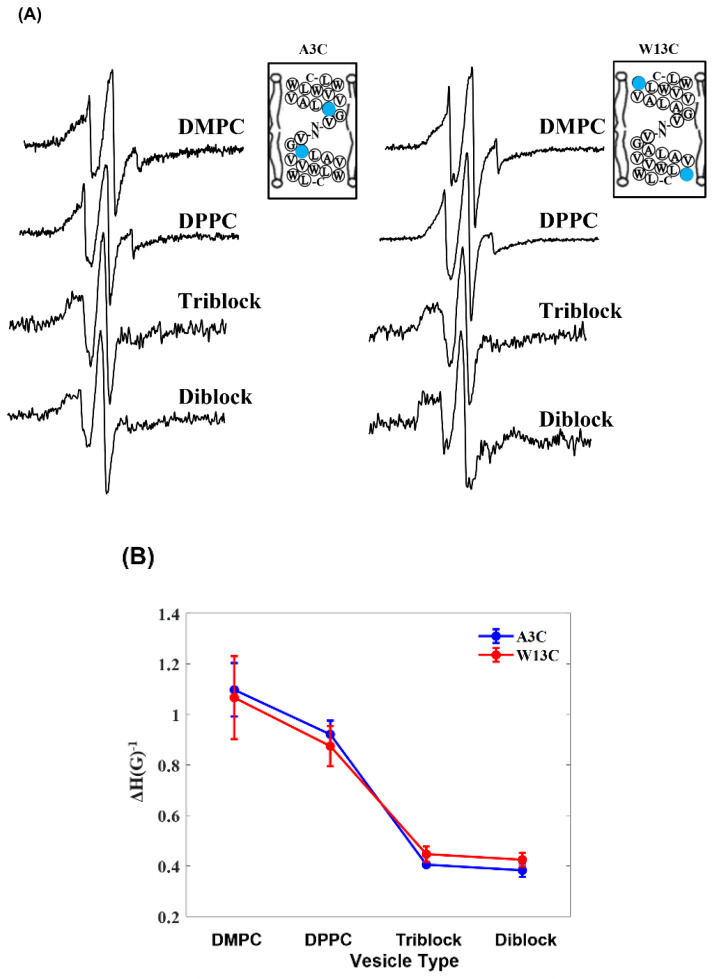
(**A**) CW-EPR spectra, collected at 298 K, for gramicidin A mutants: A3C and W13C incorporated into DMPC liposomes, DPPC liposomes, triblock polymersomes, and diblock polymersomes. Both mutants are located inside the membrane, as shown by the insets depicting the amino acid sequence and head-to-head dimer formation. The blue dots represent the sites mutated for the addition of a spin label. (**B**) Inverse central linewidth (ΔH^−1^) of EPR spectra as a function of the vesicle types containing the mutants. The error bars were determined from three repeated measurements.

Gramicidin A was incorporated into polymersomes to characterize its formation in a thicker membrane via EPR spectroscopy. Figure 2A shows the CW-EPR spectra for liposomes composed of either DMPC or DPPC as well as polymersomes composed of either triblock or diblock copolymers. Two different gramicidin A mutants were used, A3C and W13C, for spin labeling. Minimal lineshape differences were seen between the two sets of spectra, as both mutants lie within the membrane and would behave similarly in each membrane variety.

Slight peak broadening was observed in the spin-labeled gramicidin A EPR spectra in DMPC and DPPC spectra. Both lipids are fully saturated, but DMPC has a shorter acetyl chain length. The triblock polymersome and diblock polymersome spectra exhibited significant lineshape changes when compared to the liposomes. Previous studies have shown that thick block copolymer membranes will either contract around the incorporated membrane protein or arrange smaller block copolymer chains around the protein while longer chains surround it to stabilize the membrane [36]. This would explain the differences seen in the spectra as a compressed membrane around the membrane protein would likely cause additional spin-label-restricted motion, resulting in linewidth broadening. There is also slight line broadening observed between the triblock and diblock polymersome samples, which can be attributed to the thicker membrane and higher lateral pressure in the diblock polymersomes [34]. This broadening is quantified in Figure 2B. The inverse central linewidth is plotted for each vesicle type in both mutants. This value decreases as the membrane thickness of the vesicle increases, indicating peak broadening in the spectra as the membrane thickness increases.

**Figure 3:**
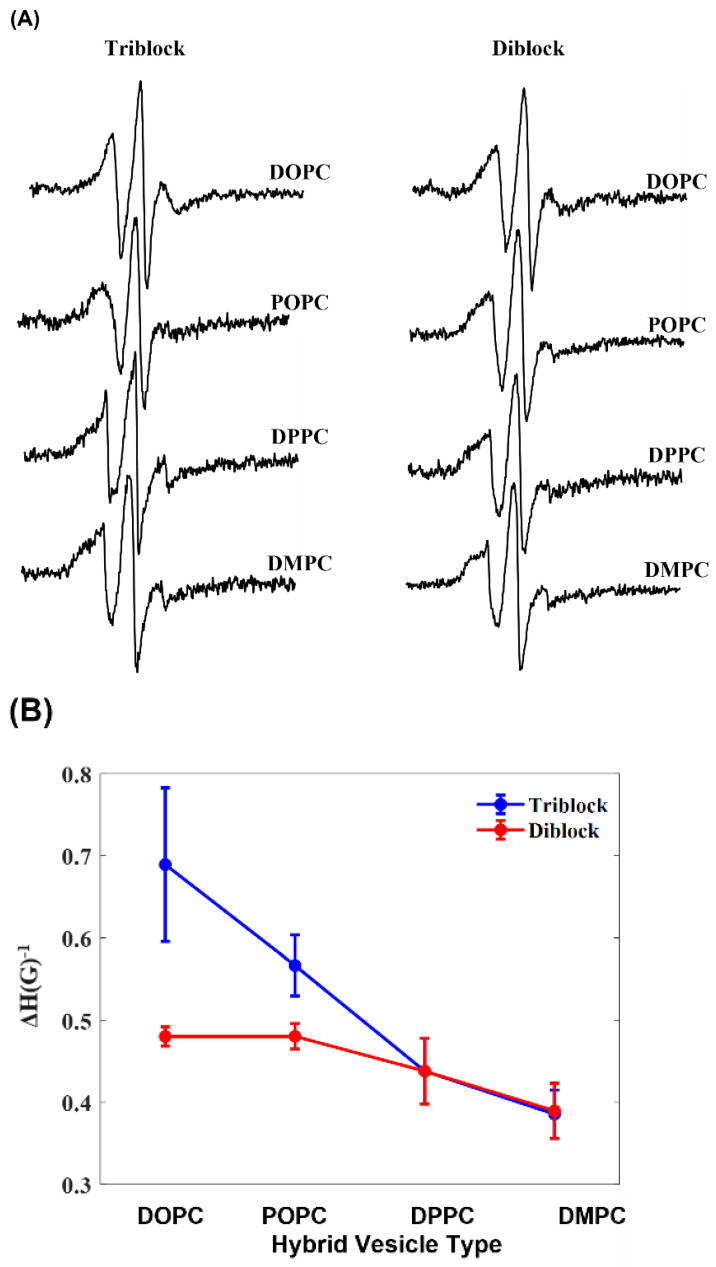
(**A**) CW-EPR spectra, collected at 298 K, for spin-labeled gramicidin A (A3C) incorporated into hybrid vesicles. Hybrid vesicles were composed of a mixture of either triblock or diblock copolymers and either DOPC, POPC, DPPC, or DMPC lipids. (**B**) Inverse central linewidth (ΔH^−1^) of EPR spectra as a function of the vesicle types containing the mutants. The error bars were determined from three repeated measurements.

Spin-labeled gramicidin A was also investigated in hybrid vesicles, shown in Figure 3A. These vesicles were made using either triblock or diblock copolymers and either DOPC, POPC, DPPC, or DMPC lipids. The lipids used have varying degrees of saturation, with DOPC being fully unsaturated, POPC having one unsaturated acetyl chain, and both DPPC and DMPC being fully saturated. There is an increase in linewidth broadening with an increase in saturation, as saturated lipids are less fluid than unsaturated lipids due to a lack in double bonds [3]. There is also additional peak broadening between triblock and diblock samples. Diblock copolymers are thicker and have a higher lateral pressure and lower lateral diffusivity in the bilayer than the triblock copolymers, resulting in the increase in linewidth [12]. This is quantified in Figure 3B. The inverse central linewidth is plotted for each hybrid vesicle type. This value decreases as the lipids become more saturated, indicating peak broadening in the EPR spectra.

The differences seen between the polymersome and hybrid vesicle EPR spectra could be due to the differences in the composition of the systems. Broader peaks were seen in the pure triblock and diblock polymersomes compared to the hybrid vesicles made with the same block copolymers. Statistically, the hybrid vesicles are variable in composition, with there being a random distribution of lipids and block copolymers within each vesicle. There is also the random chance of some vesicles being composed of 100% lipid or polymer. Although it is expected that hybrid vesicles have more pressure within the membrane, due to the differences in the thickness of the hydrophobic core of lipids and block copolymers [16], the random distribution of not only the lipids and polymers but also the incorporation of the peptide could cause differences in peak broadness. Our results show larger error bars on the inverse central linewidth (ΔH^−1^) plots for the samples that have a narrower EPR spectral linewidth when compared to the samples that have a broader linewidth. This variation in the error bars may be due to the fact that the spin-labeling sites for these samples are more dynamic and more flexible, causing variation in the stability of the lipid complex. Another contribution may be due to the characteristic of EPR spectral measurements at different linewidths [43]. Contributions might also be due to different lipid saturation levels and different lengths of the carbon acyl chain of the lipid causing variation in the stability of the lipid vesicles. Also, the variation in the sample preparation quality for different lipid samples may have contributed to the variation in the error bars.

**Figure 4:**
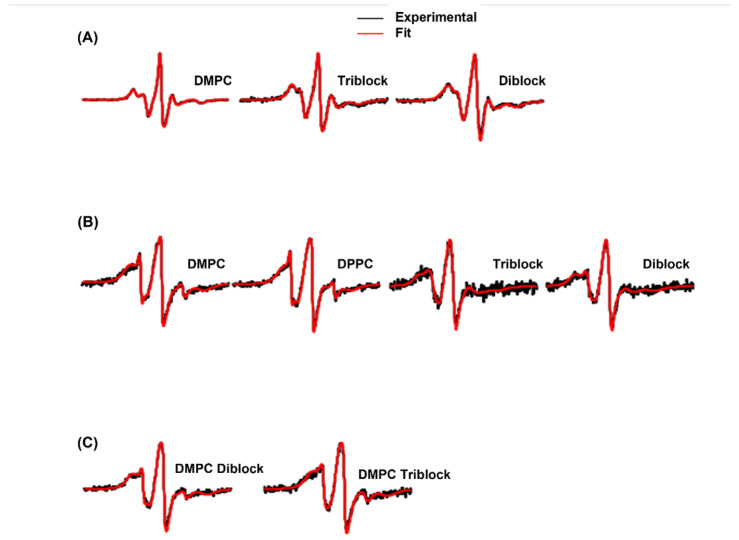
EPR spectral simulation for (**A**) 16:0 5-doxyl PC spin-labeled lipids in DMPC, triblock, and diblock vesicles; (**B**) gramicidin A mutant A3C incorporated into DMPC, DPPC, triblock, and diblock vesicles; (**C**) gramicidin A mutant A3C incorporated into hybrid vesicles composed of DMPC/triblock and DMPC/diblock. Experimental data are in black and the fitted data are in red.

The two motional components seen in the CW-EPR spectra were characterized by non-linear least squares (NLSL) simulations, shown in Figure 4. In Figure 4A, representative CW-EPR spectra of 5-doxyl PC incorporated in vesicles composed of DMPC, triblock, or diblock copolymers are shown. The simulation result indicates that the motion of the acyl chain at position 5 is slower, with a rotational correlation time of 10.3 ns and a relative population of 70% (rigid component) and 5 ns of 30% (motional component) in DMPC when compared to the block copolymers. The spectra of the triblock polymersomes showed a rotational correlation time of 7.3 ns with a relative population of 59% (rigid component) and 7 ns of 41% (motional component). Similarly, the diblock polymersomes showed a rotational correlation time of 7.3 ns with a relative population of 60% (rigid component) and 6.8 ns of 40% (motional component).

Figure 4B shows the representative CW-EPR data for the gramicidin A A3C mutant incorporated into DMPC, DPPC, triblock, and diblock vesicle samples. The polymersomes showed a rotational correlation time of 19.1 ns of 53% and 18.3 ns of 74% (rigid component) and 1.4 ns of 47% and 0.6 ns of 74% (motional component) for triblock and diblock samples, respectively. This was slower when compared to the lipid vesicles, which had a rotational correlation time of 10.1 ns of 54% and 10 ns of 42% (rigid component) and 0.4 ns of 46% and 0.2 ns of 58% (motional component) for DMPC and DPPC, respectively. Figure 4C shows the representative CW-EPR data for the gramicidin A A3C mutant incorporated into hybrid vesicles composed of DMPC and either triblock or diblock copolymers. The hybrid vesicles showed a rotational correlation time of 14.5 ns of 92% and 10.8 ns of 92% (rigid component) and 0.3 ns of 10% and 0.3 ns of 8% (motional component) for triblock and diblock hybrids, respectively. The slower motion of the spin labels in the polymersome samples is consistent with the spectral linewidth data and the lineshape changes seen in the CW-EPR spectra. This further suggests that the thicker block copolymer membranes compress around the peptide, causing more restricted spin label motion.

## 4. Conclusions

Gramicidin A was successfully incorporated in both polymersomes and hybrid vesicles and subsequently characterized using CW-EPR spectroscopy. The CW-EPR data revealed spectral line broadening for spin-labeled gramicidin A incorporated in triblock and diblock polymersomes when compared to gramicidin A in liposomes. EPR spectral lineshape broadening was also observed in the hybrid vesicles when compared to liposomes. This lineshape change suggests that the thicker membrane in triblock and diblock polymersomes compresses around the incorporated peptide and restricts the motion of the spin label. This work provides valuable information for the study of membrane proteins, using polymer polymersomes as a biomimetic, via CW-EPR spectroscopy.

## Data Availability

Dataset available on request from the authors.
